# Patients' Perception of Comfort Facilitators During Hemodialysis Procedure: A Qualitative Study

**DOI:** 10.5812/ircmj.19055

**Published:** 2014-07-05

**Authors:** Seyed Reza Borzou, Monireh Anosheh, Esa Mohammadi, Anoshirvan Kazemnejad

**Affiliations:** 1 Department of Nursing, Faculty of Medical Sciences, Tarbiat Modares University, Tehran, IR Iran; 2 Department of Statistics, Faculty of Medical Sciences, Tarbiat Modares University, Tehran, IR Iran

**Keywords:** Renal Dialysis, Comfort, Perception, Qualitative Research

## Abstract

**Background:**

The patients receiving hemodialysis spend a lot of their lifetime in the hemodialysis departments, which is an unpleasant experience. Therefore, some interventions are necessary to relieve this experience.

**Objectives:**

The current study aimed to explore the hemodialysis patients' perceptions of comfort facilitators during the hemodialysis procedure.

**Patients and Methods:**

This study was conducted by a qualitative content analysis approach. Twenty four patients receiving hemodialysis participated in this study by purposive sampling. The sampling was over when the data saturation occurred. The semi-structured interviews were applied as the main data gathering tool. The data analysis was conducted by conventional qualitative content analysis in eight phases.

**Results:**

Three themes emerged: The presence of competent nurses, the delightful presence of the others, and coping with comfort obstacles. Each theme consisted of some categories.

**Conclusions:**

It seems that to achieve the patients' comfort during the hemodialysis procedure, the health care teams, hospitals in charge and the patients themselves have to do their best to provide the patients' comfort.

## 1. Background

Chronic kidney disease includes a range of different pathological processes that cause the irreversible loss of kidney function (1). These patients` survival depends on renal replacement therapy, such as dialysis and transplantation (2). Among these treatment methods, hemodialysis is a common and successful one to control renal diseases (3). In the USA, 91.9% of renal patients underwent hemodialysis treatment in 2009 (4). In Iran, hemodialysis is also the most common renal replacement therapy (5), and 50% of the patients undergo this treatment (6). These patients need to be constantly present in the hemodialysis centers. As they spend three sessions a week, each three to five hours (on average three and a half hours) under dialysis treatment (7), they should feel comfortable and relaxed.

Comfort is a basic concept in nursing (8), a nursing outcome and practice, a basic human need, an inherent procedure and need throughout the life (9). It is the patients and their families highest demand and an important element of nursing care (10). This concept has been a concern in nursing to the extent that Apostolo has offered a specific theory to explain it; he defined it as the ability to meet basic human needs to achieve relief, ease and transcendence in physical, psychological, cultural, social and environmental dimensions (11).

According to Kolcaba's theory, relief is a state achieved by the individual, right after meeting his needs. Ease is the sense of comfort and contentment in the patient, subsequent to alleviating his needs, and transcendence is the state when the individual can achieve goals beyond the feeling of pain and discomfort by overcoming them (11, 12). Also, in this theory, comfort is defined as possessing affective emotional and physical elements by the individual who has experienced it (13), meaning that the concept of comfort is highly subjective, vague, and varying based on individuals` thoughts and ideas (10). In Keesler's view, the concept of comfort is excessively subjective, in the sense that what is comfortable for a patient may be different for another; or an action might be comforting for a care-giver, but not so for a patient, etc. (14). Therefore, the identification and practice of comfort measures are important objectives of the art of nursing and caring, which are also important in patients receiving hemodialysis. They need ease and comfort during the dialysis process, because of the long home-center distance, the constant presence in the centers, frequent trips to hospitals, having three dialysis sessions per week each lasting three and a half hours, painful venipuncture procedures, long in-bed resting, disease-related insomnia, and problems related to technical issues (7, 15-20).

Different factors play roles in comforting the patient. Identifying, exploring and recording these factors help true understanding of the existing capacities and facilities. Therefore, it is necessary to consider important caring aspects as a sense of tranquility for the patients. This goal cannot be achieved unless the patients and their experiences are used, since studies show that nurses do not know their patients and their expectations well, and assessing them based on their own views, most of the times they do not examine the patients’ needs (21).

As it is difficult to study human phenomena through numerical and quantitative values and mathematical formula, research methods should be employed that seek to know deep and inner realities of human beings; qualitative studies can be effective in this respect (22, 23). According to the preceding remarks, the long-standing educational and clinical experiences of the researcher, his numerous publications in accredited domestic and international journals about patients receiving hemodialysis, the multi-dimensional and the subjective nature of the concept of comfort it should be noticed in this respect that no research was found in Iran.

## 2. Objectives

The current study aimed to explore the patients` perception of the facilitators in the process of hemodialysis.

## 3. Patients and Methods

### 3.1. Design

This qualitative research was conducted by a conventional qualitative content analysis.

### 3.2. Setting and Participants

The research setting was the hemodialysis centers of two teaching hospitals in Hamadan city, Iran.

### 3.3. Data Collection

The data were collected through semi-structured interview from January to July 2013. The interviewer was the first author in the current paper experienced both as a clinical trainer and dialysis nurse for 20 years. The interview began with a general open question: “Please speak to me about hemodialysis” to win the patients’ trust; then probing questions followed: “Please let me know how you are feeling during the hemodialysis, has there been a time during the hemodialysis that you have enjoyed more comfort? Please explain that situation”.

To clarify the participants’ understanding, follow-up questions were also asked based on the information provided by them. The interviews were face–to-face, with the length of 45 to 60 minutes, depending on the physical status and the inclination of the participants. A purposive sampling with the homogenous approach was used to select the participants and continued until data saturation, when no new data emerged. The inclusion criteria were irreversible renal disease with at least two times hemodialysis per week, being conscious and able to talk, lack of disability and other physical and psychological diseases.

Obtained data from analysis of each interview was a guide for the next interview; therefore, sampling continued until data saturation was achieved with 20 participants; however, interviewing continued with four other participants for further assurance. As no new codes were derived in these interviews, totally 24 participants were interviewed.

### 3.4. Data Analysis

In fact, data analysis was conducted parallel with data collection. The data analysis was performed using a conventional content analysis method, and the data processing was conducted with an 8-stage systematic and transparent method.

In the first stage, the interviews were written down and arranged for a qualitative content analysis. In the second stage, all the texts were read several times before encoding in order to become fully familiar with the data, then decision was made about the coding units. In the third stage, the categories were derived inductively from the initial raw data. To determine the distinction among the categories, a constant comparison method was used. In the fourth, codes were again examined in terms of the clarity and stability of the categories definition. After the coding stage, the data were organized to prepare for the next step.

In the fifth stage, the entire text was coded. The researcher constantly controlled the coding process to ascertain the agreement among the participants and the research team members. In the sixth stage, the consistency in the text coding was controlled. In the seventh stage, the characteristics and dimensions of the categories, inter-category relations were identified and the hidden concepts were uncovered and reviewed according to the data, and meaning units were offered based on the data. In the final stage, the analysis method, the work procedures, such as coding and the applied methods, were completely reported to the research team members in order to achieve an appropriate method for the study.

### 3.5. Trustworthiness

The data trustworthiness was evaluated by credibility, transferability, and conformability (24).

To check the reliability and validity of the data, the time-integration technique was used through sampling on three occasions in the morning, in the afternoon, and at night. To make sure of the homogeneity of the extracted data, the interview texts were given to the participants to confirm the accuracy of the extracted concepts. To ensure data appropriateness, a maximum-diversity sampling technique was used, which assisted the appropriateness or the transferability of the findings to the others or to the audience. Also, the conformability of the findings was examined by member check. To confirm the dependability of the data, its procedures were precisely recorded and reported in order to let the others follow up the study.

### 3.6. Ethical Considerations

This study was approved by the Research Council and Ethics Committee of Trabiat Modares University of Medical Sciences, (No: D52/2087). Research ethical principles such as informed consent, anonymity, confidentiality, and the participants’ freedom to withdraw from the study were observed. Also, before the onset of the interviews, participants were informed about the objectives of the study, the confidentiality of the information, and the recording of interviews and their written consent for participation was obtained.

## 4. Results

Out of 24 participants in study, 570 primary codes, 20 subcategories, eight categories and three themes were concluded. The mean age of participants was 40.37 ± 14.23 and mean length of hemodialysis was 3.91 ± 3.23 hours (Table 1). Three themes were derived from the interviews:

1. The presence of competent nurses,
2. The delightful presence of the relatives, and
3. coping with comfort-obstacles (Table 2).

**Table 1.** Characteristics of the Patients Under Study ^a^

Variable	Number (%)
**Sex**	
Female	9 (37.5)
Male	15 (62.5)
**Marital status**	
Married	14 (58.33)
Single	6 (25)
Separated	3 (12.5)
Widow	1 (4.17)
**Occupation**	
Housekeeper	5 (20.83)
Employed	5 (20.83)
Unemployed	8 (33.34)
Retired	6 (25)
**Educational level**	
Primary	10 (41.66)
Secondary/diploma	7 (29.17)
University	7 (29.17)

^a^ Data are presented as No. (%).

**Table 2.** Major Themes and Categories

Themes	Category
**The presence of competent nurses**	
	Having human relationship
	Having professional responsibility
	Having knowledgeable, skilled, expertise
**The Delightful presence of others**	
	The Positive effects of peer
	Comforting presence of family and friends
**Coping with comfort-obstacles**	
	Religious coping
	Positive thinking
	Attempting to pass the time faster

### 4.1. The Presence of Competent Nurses

This theme includes the categories of possessing human relationship, professional responsibility, knowledgeable, skills and expertise. The majority of the patients said that a convenient hemodialysis requires the presence of competent, knowledgeable, skilled, experienced, and accountable nurses with good personal relationship.

The participants’ experiences showed that when the patient was dealt with affably, he felt comfortable. A 33-year- old man with 3.5 years of hemodialysis experience said: “Look! This is not just about the patient. Everyone likes to be treated cordially wherever they go; you know, be accepted”.

An aspect of caring attitude noted by some participants was accountability during the operation. A 33-year-old man with four years of hemodialysis experience stated: “They (nurses) have a vital role in our comfort. In my opinion, it matters a lot that the patient feels comfortable with the nurse during the hemodialysis; and the nurse should really be accountable, as the life of the patient connected to the machine resides out of his body, and it is always likely that one of the tubes has a hole”.

The nurse’s skills, in particular, in technical issues are very important to give the patients the feeling of comfort. The incompetence of the nurses, in particular, in venipuncture was among the complaints of some of the participants. A participant with 3.5 years of dialysis experience said: “They sometimes used seven to eight angiocatheters until finally one of them worked”.

The working experience of the nurses, not being a novice, was also comforting for the patients. A 25-year-old woman with eight years of hemodialysis experience stated: “If the nurses are good and experienced, of course most of them are and it takes the beginners a while to become skilled, we will then be comfortable”.

Having knowledge and expertise was also an issue pointed to by the participants. A participant with four years of hemodialysis experience said: “The issue of expertise is at stake here. In my viewpoint, a good nurse should be knowledgeable in terms of expertise, clever, courageous which means risk-taking, and act professionally in her work”. The issue of expertise was so important for the patient that he took it as an essential feature to employ the nurses: “A great deal of care and accuracy should be exercised in employing the nurses, it means highly expert people should be employed”.

### 4.2. The Delightful Presence of the Others

This theme includes the categories of the positive effects of peer, comforting presence of family and friends. This theme addresses the fundamental role played by the presence of other similar patients, friends, and the family for the comfort of patients during the hemodialysis. The patients would feel tranquility from the positive presence of their peers. A 51-year-old woman stated: “A good roommate is a comfort. I am often with ladies; well, they talk and we feel comfy". Visitors and companions were also highly effective in heartening and gratifying the patient. A 30-year-old woman with five years of hemodialysis experience said: “Visitors talk with the patient, so they feel good; we do not have any amusement here to engage ourselves with”. Among this group, family members were the most important comforting element for the patients. A 58-year-old man said: “I often come with a family member for the hemodialysis. They come with me whenever I come, which makes me feel comfortable during the hemodialysis”.

### 4.3. Coping With Comfort-Obstacles

This theme describes the patient’s behavior and practice for attaining comfort during the dialysis, which includes the categories of religious coping, positive thinking and attempting to pass the time faster. Believing in God’s power and greatness is the source of heartening and comfort for the patients. A 50-year-old man said: “When I look at the machine, I stare at it. In fact, we realize God’s power. I personally do not claim to be religious; no! But I sometimes realize God’s power, as He has put such a small kidney in the human being’s body; on the other hand, you see such a big machine is doing the work of the kidney; this very fact relaxes me; it truly makes me relieved; I become totally relaxed and enjoy a peace of mind.

Some patients would attain comfort and tranquility by reciting Salavat (Hail the Prophet Mohammad): “I sometimes recite Salavat for myself. I feel relax and cool by reciting Salavat”.

Positive thinking and creating a positive attitude in the patient towards hemodialysis make him feel more comfortable; as a 33-year-old male participant claimed: “Whenever I am in a good mood, and look at everything positively, I feel relax".

### 4.4. Attempting to Pass the Time Faster

The patients considered one of the serious problems of hemodialysis the time-consuming nature of its sessions, holding that tolerating four hours of hemodialysis was very difficult for them. They maintained that they gradually learned to pass the time faster by performing different activities, such as speaking with the other patients, sleeping, listening to the radio and music, and watching TV and video movies. A 20-year-old woman said: “Look! When nothing is available, I talk with visitors or patients. This helps a bit to slightly reduce these four hours”.

A participant stated listening to music on the radio and mobile: “For example, when I come here, I listen to music on my mobile, or listen to the Quran, or read a book; I have to somehow pass these three hours”. Watching TV and studying were among other methods to pass the time during the hemodialysis: “I watch the TV to get busy and pass the time, if I bring a book, I’ll read it; otherwise, if I couldn’t make myself busy with something, I’ll close my eyes to fall asleep".

## 5. Discussion

The results of the current study showed the interaction between the three human factors as nurses, the patients and the people surrounding the patients. The competent nurse with the professional practice, people with the positive presence, and the patient who use coping mechanisms can facilitate the hemodialysis and provide comfort. Patients, expect nurses to have appropriate conduct. Fakhr-Movahhedi et al. observed, in their qualitative study, that nurses` behavior is comforting patients during their acute needs. Nurses’ friendliness, consoling, respecting the patient, empathy and patience at facing the patients’ needs make them feel at home, which in turn comforts the patients (25). The results of this study seem to be in line with those of the current study. Because of the chronic nature of the disease and the dialysis treatment, many changes and difficulties could occur in their occupation, family life, financial status, diet, and etc. These problems along with regular three-time-a-week hemodialysis request a more frequent patient-nurse relationship (26).

Another aspect of caring attitude noted by some participants was accountability. Farber quotes that all professional people working in the treatment centers should have accountability, which should reveal itself in the nurse responsible behavior (27). Mohammadi in his study on heart-surgery patients found that accountability was among the concepts that concerned the patients (28).

Knowledge, experience, and nimbleness of the nurses were among the issues emphasized by the participants. Han quotes that the demographic characteristics of nurses, and their knowledge and experience affect patients’ satisfaction (29). The study by Mohammadi also showed that the majority of patients who were directly cared by a competent nurse enjoyed peace of mind and comfort (28). The importance of the above issues is due to the fact that the hemodialysis center, compared to other centers, is a fully technical and specialized one. Therefore, a nurse who is working there should also possess technical expertise, precision in her work, adequate information about the patient, dialysis treatment, and hemodialysis complications (26).

Another finding of this study concerned the positive comforting effect of the peers. Studies have shown that peer group support is effective for renal patients and for patients who are undergoing long hemodialysis treatment (29); however, the important and fundamental point here is the presence of patients with a positive and happy spirit, which comforts not only the person but also the others.

The participants also played the role of visitors during the hemodialysis. The presence of visitors beside the patients’ bed heartens these patients. Visitors play an inspiring role in the absence of the nurse by speaking with the patient and caring for him. But, hospital policies are different in this regard. Many centers totally prevent visiting because of over crowdedness, necessity of quick actions in emergency situations, observing the privacy of the patient, and preventing noise and infection. However, some centers allow their presence for their assistance and support during the treatment, creating a friendly environment, the convenience in training the families, and assisting in setting the bed, etc. (30).

Accordingly, based on the findings of the conducted studies, and due to the importance of the presence of these people, and their advantages and disadvantages for patients, the accompaniment of at least one person is necessary. The role of the family was also emphasized by the patients. Bellou et al. mention that family has a significant role in patients’ treatment at hospitals. They can assist patients who are under treatment in hospitals by offering effective psychological and emotional supports (31). Bellou’s view seems to be highly practical for short-term hospitalized patients. The families of the patients who receive hemodialysis cannot constantly accompany them, despite patients' tendency. The patients claimed that they should spend at least four hours in each session which is pretty long.

Another finding of the study was the issue of easy coping with obstacles, denoting that patients were active in the process of achieving comfort during the hemodialysis. The patients believed that, despite many problems of hemodialysis, they felt comfortable because of positive thinking. Many studies have shown that a positive attitude is vital to maintain motivation (32).

The present study showed that believing in God’s greatness and the performance of religious rituals such as praying and reliance on God have been the effective methods used by hemodialysis patients, which are mainly related to religious aspects of the people’s understanding of comfort and their culture. In a study by Yousefi et al. in Iran, In Search of God was a theme related to the calmness of the hospitalized patients (33). The central variable in Walton’s qualitative study conducted based on grounded theory to explore the meaning of spirituality among American Indians who were under hemodialysis, was the notion of prayer warriors. Praying played a significant role in this research (34).

It is obvious that hemodialysis patients should spend three 4-hour dialysis sessions per week, which takes a large portion of their time and makes them face a boring experience (15); therefore, the patient uses different approaches to pass this time. A research by Asgari et al. showed that patients would study and sleep to pass the time more quickly (35); therefore, according to the results of the present study and its consistency with the other performed studies, providing adequate books, journals, and newspapers in dialysis centers should be considered to let patients read, get relieved, and entertained.

The current study results indicated that the treatment team, hospital officials and the patient himself should do their utmost to let hemodialysis patients achieve comfort, which is not possible without the intervention of nurses and hospital officials. Nurses can play a significant role in comforting these patients because they have the most direct contact with them and are able to identify and provide comforting elements. Nurses play a key role in the treatment team. They can form suitable relationship, assist to create positive attitude in the patient, and provide an appropriate caring context for them. Besides, to a large extent officials can help patients achieve their aims by providing welfare, cultural and ritual facilities, and allowing the presence of family members and visitors along with observing the others’ rights and security issues.

The limitation of this study, similar to the other qualitative studies, was that the findings could not be generalized to the target population. Thus; the researchers in the current study tried their best to achieve the maximum reliability. 

To the best of researchers’ knowledge, one of the strong points of the current study was its novelty in Iran. Another strong point of the study was the difficulties of the patients’ interviews in such a physical, psychological and emotional status, which involved precise communication skills.
